# Prognostic value of coronary calcification detected via non-electrocardiogram-gated computed tomography in patients with cardiovascular disease: A retrospective cohort study

**DOI:** 10.1016/j.ijcha.2024.101560

**Published:** 2024-11-23

**Authors:** Tomitaka Wakaki, Yusuke Takagi, Yuto Ono, Ryosuke Kato, Ken Abe, Hiroyuki Watanabe

**Affiliations:** Department of Cardiovascular Medicine, Akita University Graduate School of Medicine, Japan

**Keywords:** Agatston score, Cardiovascular diseases, Electrocardiography, Neoplasms, Prognosis

## Abstract

**Background:**

The correlation between coronary artery calcification (CAC) detected via electrocardiogram-gated computed tomography (ECG-gated CT) and future cardiovascular events has been well-established. Non-ECG-gated CT is simple and widely used, making it suitable for screening. However, the correlation between CAC observed via non-ECG-gated CT and cardiovascular and non-cardiovascular events remains unclear. Therefore, we examined the association between coronary calcification detected via non-ECG-gated CT and prognosis.

**Methods:**

This non-randomized, retrospective cohort study included 353 consecutive patients with cardiovascular diseases (male/female 229/124; mean age, 68.6 ± 12.7 years) who underwent non-ECG-gated CT between October 1, 2017 and May 31, 2021. Correlations between the Agatston score and cardiovascular and non-cardiovascular events were evaluated. The Agatston scores were divided into three tertiles (low, intermediate, and high) and compared. The primary endpoint was composite cardiovascular events, including cardiac death, myocardial infarction, hospitalization for congestive heart failure, stroke, and unplanned cardiac surgery. The secondary endpoint was composite non-cardiovascular events, including non-cardiovascular death, cancer development, and hospitalization for a non-cardiovascular worsening event.

**Results:**

During the median follow-up period of 16.9 (interquartile range, 2.2–38.6) months, 83 patients reached the primary endpoint, while 81 patients reached the secondary endpoint. Kaplan–Meier analysis indicated that patients with high Agatston scores had a significantly higher incidence of cardiovascular and non-cardiovascular events than those with low Agatston scores (*p* < 0.001).

**Conclusions:**

In this study, the Agatston score obtained using non-ECG-gated CT predicted cardiovascular and non-cardiovascular events. Non-ECG-gated CT can be easily performed, aiding early detection in patients with high event rates.

## 1 Introduction

Coronary artery calcification (CAC) scores, including the Agatston score, are useful tools for predicting coronary artery stenosis and cardiovascular events [1], [2], [3], [4]. These scores are defined using electrocardiogram (ECG)-gated computed tomography (CT). Although non-ECG-gated CT is simple, widely used, and suitable for screening, its predictive value compared with that of ECG-gated CT remains unclear [5], [6], [7], [8], [9]. Furthermore, although an association between whole-body calcification volume and future adverse events has been reported, the relationship between CAC scores determined using non-ECG-gated CT and non-cardiovascular events has not been thoroughly investigated [10], [11], [12], [13].

In this study, we assessed the following. First, we examined the correlation between CAC scores derived from non-ECG-gated CT and both cardiovascular and non-cardiovascular events. Second, we investigated whether a significant association existed between CAC scores from non-ECG-gated CT and non-cardiovascular events. Through these assessments, our aim was to determine the clinical potential of CAC scores from non-ECG-gated CT.

## 2 Methods

### 2.1 Study population

In this retrospective, single-center, retrospective cohort study, we analyzed the data of 353 consecutive patients with cardiovascular diseases (male/female 229/124; mean age, 68.6 ± 12.7 years) who underwent non-ECG-gated CT between October 2017 and May 2021. Data were collected from electronic hospital records. No patients were excluded, and all patients underwent coronary angiography, resulting in a comprehensive dataset.

### 2.2 Data collection

Patient information including age, sex, medical history, history of cancer, coronary risk factors (hypertension, dyslipidemia, diabetes mellitus, and smoking), diagnosis after catheterization, medication details, echocardiographic data (ejection fraction), blood test data (creatinine), clinical symptoms, and clinical events during the follow-up period was obtained from electronic medical records. Non-contrast CT images were acquired using 64-slice multidetector CT scanners with a 5-mm slice thickness. The Agatston score was calculated using a workstation (Synapse Vincent; Fujifilm, Tokyo, Japan), and calcification scores were calculated semi-automatically. The Agatston score was defined based on a 3-mm slice thickness; for scans obtained with a 5-mm CT slice thickness, the score required multiplication by a factor of 1.67. This correction was crucial for ensuring methodological consistency and preserving the accuracy of the Agatston score.

### 2.3 Clinical data and endpoint

Hypertension was defined as systolic blood pressure ≥ 140 mmHg or diastolic blood pressure ≥ 90 mmHg at presentation, diagnosis of hypertension by a physician, or use of antihypertensive medication. Diabetes mellitus was defined as fasting blood glucose ≥ 126 mg/dL, diagnosis of diabetes by a physician, or use of antidiabetic medication. Dyslipidemia was defined as total cholesterol concentration > 220 mg/dL, diagnosis of dyslipidemia by a physician, or use of lipid-lowering medication. Patients were considered smokers if they had smoked previously. The Japanese glomerular filtration rate (GFR) equation was used to calculate the estimated GFR (eGFR) as follows: eGFR (mL/min/1.73 m^2^) = 1.94 × [creatinine]^–1.094^ × age^–0.287^ (with the result being multiplied by 0.739 in women) [14]. Chronic kidney disease was defined as eGFR < 60 mL/min/1.73 m^2^ at admission. Patients with atrial fibrillation were defined as those presenting with atrial fibrillation as determined via admission electrocardiography and those with a history of paroxysmal atrial fibrillation. Coronary artery stenosis was defined as severe stenosis > 70 % according to coronary angiography, following the American Heart Association stenosis classification [15].

The primary endpoint was that of composite cardiovascular events, including cardiac death, nonfatal myocardial infarction, hospitalization for congestive heart failure, stroke, and unplanned cardiac surgery. All deaths not attributed to non-cardiac causes were presumed to be cardiac in origin [16]. Unplanned cardiac surgery included all cardiovascular procedures not scheduled at the time of coronary angiography during the first hospitalization, including bypass surgery, valve replacement, and major vessel replacement. The definition of stroke included both cerebral infarction and cerebral hemorrhage. Congestive heart failure hospitalization was defined as admission for congestive heart failure.

The secondary endpoint was defined as composite non-cardiovascular events, including non-cardiovascular death, cancer development, and hospitalization for a worsening event unrelated to a cardiovascular event.

Non-cardiovascular death was defined as death from non-cardiovascular causes. Cancer development was defined as cancer occurrence during the observation period. Hospitalization for a worsening non-cardiovascular event was defined as hospitalization for an adverse non-cardiovascular event, including infection and bleeding.

### 2.4 Statistical analysis

Continuous variables are presented as medians and interquartile ranges (IQRs) or means and standard deviations, whereas categorical variables are expressed as numerals and percentages. Group comparisons were performed using one-way analysis of variance for continuous variables with normal distributions, the Kruskal–Wallis test for continuous variables with non-normal distributions, the chi-square test for categorical variables, and the log-rank test for survival curves. Survival free from cardiovascular and non-cardiovascular events was assessed using the Kaplan–Meier method. Statistical significance was set at *p* < 0.05.

Univariate and multivariate Cox proportional hazard models were used to identify variables associated with cardiovascular and non-cardiovascular events. Variables that exhibited statistical significance or a trend (*p* < 0.1) from the univariate Cox model were fitted into the multivariate analysis using the forced entry method, with variables incorporated in order of their *p*-values (starting from the lowest). The proportional hazards assumption for the Cox model was evaluated using a log-minus-log plot. Furthermore, the interaction between risk strata and predefined clinical subgroups regarding their effects on cardiovascular and non-cardiovascular events was assessed using Cox models. The analysis accounted for various confounding factors, ensuring a robust examination of the associations. Statistical analysis was performed using SPSS Statistics version 29.0 (IBM Corp., Armonk, NY, USA), with all *p*-values being two-tailed. Multiple imputation was not performed because of concerns regarding potential misinterpretation in cases of missing data.

## 3 Results

### 3.1 Patient characteristics

Between October 2017 and May 2021, we enrolled 353 consecutive patients with cardiovascular diseases (male/female 229/124; mean age, 68.6 ± 12.7 years) who underwent non-ECG-gated CT. All patients underwent coronary angiography and received interventions as needed. The principal diagnoses after cardiac catheterization were ischemic heart disease (182 patients), valvular disease (59 patients), cardiomyopathy (36 patients), arrhythmia as the primary diagnosis (20 patients), vasospastic angina (11 patients), arteriosclerosis obliterans (9 patients), pulmonary hypertension (7 patients), and other cardiovascular conditions (29 patients). The median Agatston score in all cases was 891. The patients were divided into tertiles based on the Agatston score and categorized into three groups (low [median, 0], intermediate [median, 200], and high [median, 1601]). Table 1 lists the patient characteristics. Patients in the higher Agatston score group tended to be older, be male, and have a higher prevalence of hypertension, dyslipidemia, diabetes, history of smoking, prior myocardial infarction, renal failure or dialysis, obstructive artery disease, and pacemaker implantation. Greater severity of coronary artery disease was observed in patients with higher Agatston scores than in patients with lower scores. Additionally, lower scores were associated with a higher proportion of patients with low left ventricular ejection fraction. No significant differences in cancer prevalence or stage were observed between the groups.

**Table 1:** Baseline patient characteristics.

Agatston score (third quartile)	Low	Intermediate	High	p-value
No. of patients, n (%)	117	118	118	
Age, median (IQR), years	67 (53, 75)	72 (66, 79)	72 (65, 77)	<0.001
Male, n (%)	65 (56)	81 (69)	83 (70)	0.04
Coronary risk factor, n (%)				
Hypertension	68 (58)	88 (75)	102 (86)	<0.001
Dyslipidaemia	48 (41)	75 (65)	88 (75)	<0.001
Dyabetes mellitus	27 (24)	53 (48)	71 (61)	<0.001
Current Smoking	16 (14)	20 (17)	22 (19)	0.61
Past smoking	55 (47)	79 (67)	83 (71)	<0.001
Previous myocardial infarction	3 (3)	21 (18)	29 (25)	<0.001
Chronic kidney disease, n (%)	49 (42)	64 (54)	77 (65)	0.002
Dialysis, n (%)	1 (1)	7 (6)	22 (19)	<0.001
NYHA ≧ 3, n (%)	52 (44)	42 (36)	51 (43)	0.36
LVEF, % (SD)	58.6 (16.3)	59.6 (12.9)	58.3 (14.4)	0.79
LVEF < 40 %, n (%)	25 (21)	11 (10)	14 (12)	0.025
Atrial fibrillation, n (%)	35 (30)	26 (22)	29 (25)	0.37
Paroxysmal atrial fibrillation	12 (10)	7 (6)	11 (9)	0.46
Chronic atrial fibrillation	23 (20)	19 (16)	18 (15)	0.64
Vital signs on admission (SD)				
Systolic blood pressure, mmHg	119 (23)	121 (18)	123 (22)	0.35
Diastolic blood pressure, mmHg	71 (16)	69 (14)	67 (13)	0.06
Heart rate, /min	75 (17)	73 (17)	73 (18)	0.68
Past history, n (%)				
Pacemaker implantation	21 (18)	6 (5)	7 (6)	<0.001
Arteriosclerosis obliterans	2 (2)	8 (7)	20 (17)	<0.001
Cerebral infarction	10 (9)	13 (11)	16 (14)	0.47
Cerebral hemorrhage	1 (1)	3 (3)	3 (3)	0.56
Chronic obstructive pulmonary disease	23 (20)	35 (30)	35 (30)	0.13
History of cancer, n (%)	30 (26)	23 (20)	28 (24)	0.49
Complete response	9 (30)	7 (30)	8 (30)	1
Partial response	11 (37)	9 (39)	10 (37)	0.98
Stable disease	5 (17)	5 (22)	8 (30)	0.5
Progressive disease	5 (17)	2 (9)	1 (4)	0.26
Medication, n (%)				
ACE or ARB	70 (60)	73 (62)	77 (66)	0.63
Angiotensin receptor-neprilysin inhibitor	2 (2)	0 (0)	0 (0)	0.13
Aspirine	28 (24)	58 (49)	82 (70)	<0.001
Beta blocker	59 (50)	58 (49)	67 (57)	0.41
DOAC	30 (26)	20 (17)	19 (16)	0.13
Ezetimibe	5 (4)	20 (17)	12 (10)	0.006
Mineralocorticoid receptor antagonist	38 (33)	31 (26)	23 (20)	0.083
P2Y12 or Clopidogrel	15 (13)	46 (39)	67 (57)	<0.001
Sodium glucose transporter 2 inhibitor	7 (6)	3 (3)	8 (7)	0.29
Statin	45 (39)	80 (68)	91 (78)	<0.001
Warfarin	18 (15)	24 (20)	24 (21)	0.52
Perfoming treatment details, n (%)				
Coronary angiography	117 (100)	118 (100)	118 (100)	1
Coronary artery bypass grafting	1 (1)	7 (6)	12 (10)	0.008
Percutaneous coronary intervention	12 (10)	38 (32)	50 (42)	<0.001
Valve replacement or repair	14 (12)	13 (11)	5 (4)	0.079
Agatston score, median (IQR)	0 (0, 0)	200 (98, 361)	1601 (1076, 3125)	<0.001
CAC volume, median (IQR), mm^3^	0 (0, 0)	215 (126, 375)	1555 (1026, 2673)	<0.001
Coronary artery severe stenosis, n (%)	9 (8)	44 (37)	65 (55)	<0.001

ACE = angiotensin converting enzyme; ARB = angiotensin receptor blocker; CAC = Coronary artery calcification; DOAC = direct oral anticoagulants; IQR = inter quartile range; LVEF = left ventricular ejection fraction; NYHA = new york heart association; SD = standard deviation.

### 3.2 Long-term outcomes and factors correlated with the primary endpoint

During the median follow-up period of 16.9 (IQR, 2.2–38.6) months, 83 patients (23.5 %) reached the primary endpoint with 14 cardiac deaths, 20 nonfatal myocardial infarctions, 22 hospitalizations for congestive heart failure, 7 S, and 20 unplanned cardiovascular surgeries. The event rates in all patients were 80.7 % at 1 year, 69.1 % at 3 years, and 50.4 % at 5 years.

**Fig. 1:**
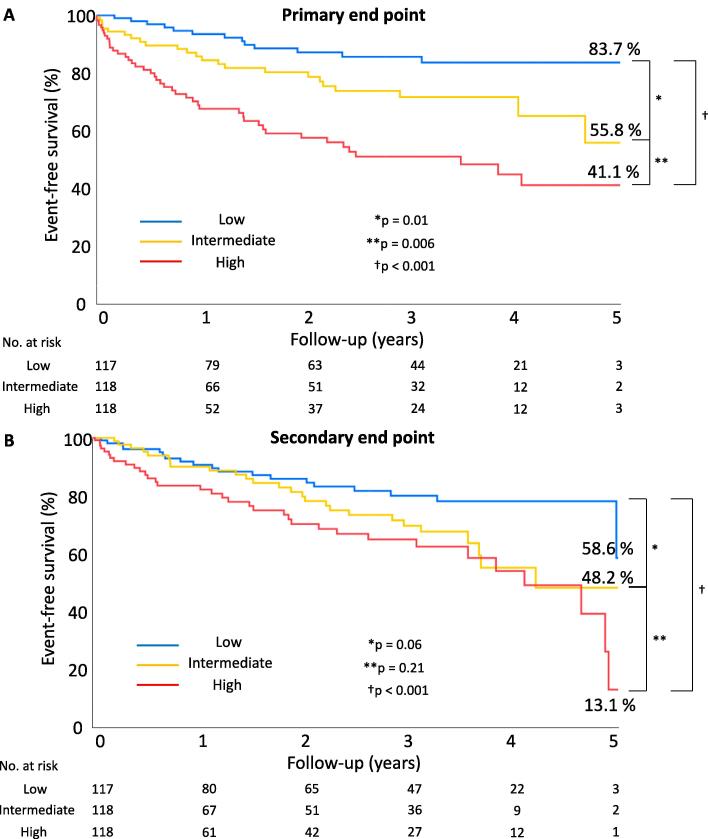
Kaplan–Meier curve analysis of the primary endpoint and the secondary end point. Kaplan–Meier analysis demonstrated the prognostic difference between patients with low, intermediate, and high Agatston scores throughout the follow-up period.

Table 2 lists patient characteristics and treatments associated with the primary endpoint, as determined via both univariate and multivariate analyses. The multivariable Cox model revealed three significant factors associated with the primary endpoint: smoking, dialysis, and Agatston score. Kaplan–Meier analysis revealed prognostic differences among patients with low, intermediate, and high Agatston scores throughout the follow-up period (Fig. 1A). A substantial increase in the event rate was observed between the low and intermediate Agatston score groups.

**Table 2:** Correlated Factors for Primary Endpoint.

	Univariable Analysis		Maltivariable Analysis	
	HR (95 % CI)	p-value	HR (95 % CI)	p-value
Age ≧ 75 years	1.48 (0.96–2.29)	0.079	1.37 (0.85–2.20)	0.19
Sex (male)	1.23 (0.78–1.96)	0.38		
Hypertension	1.50 (0.87–2.60)	0.15		
Dyslipidemia	1.54 (0.97–2.45)	0.069	1.02 (0.62–1.67)	0.94
Diabetes	1.48 (0.95–2.31)	0.84		
Past smoking	1.96 (1.16–3.31)	0.012	1.73(1.04–2.87)	0.034
Previous myocardial infarction	1.29 (0.67–2.50)	0.45		
CKD	1.91 (1.20–3.05)	0.006		
Dialysis	4.42 (2.63–7.44)	<0.001	2.80 (1.55–5.07)	<0.001
NYHA ≧ 3	1.57 (1.01–2.42)	0.043	1.49 (0.94–2.36)	0.094
LVEF < 40 %	0.78 (0.44–1.36)	0.38		
History of AF	0.99 (0.60–1.62)	0.95		
History of PMI	0.44 (0.14–1.40)	0.16		
Arteriosclerosis obliterans	3.29 (1.80–5.96)	<0.001	1.58 (0.82–3.04)	0.18
History of stroke	2.67 (1.58–4.53)	<0.001	1.62 (0.91–2.88)	0.10
History of cancer	0.61 (0.35–1.09)	0.97		
Agatston score ≧ intermediate	3.46 (1.91–6.26)	<0.001	2.33 (1.34–4.41)	0.009

AF = atrial fibrillation; CI = confidence interval; CKD = cronic kidney diesease; HR = hazard ratio; LVEF = left ventricular ejection fraction; NYHA = new york heart association; PMI = pacemaker implantation.

### 3.3 Application of the Agatston score determined using non-ECG-gated CT to clinical subgroups

**Fig. 2:**
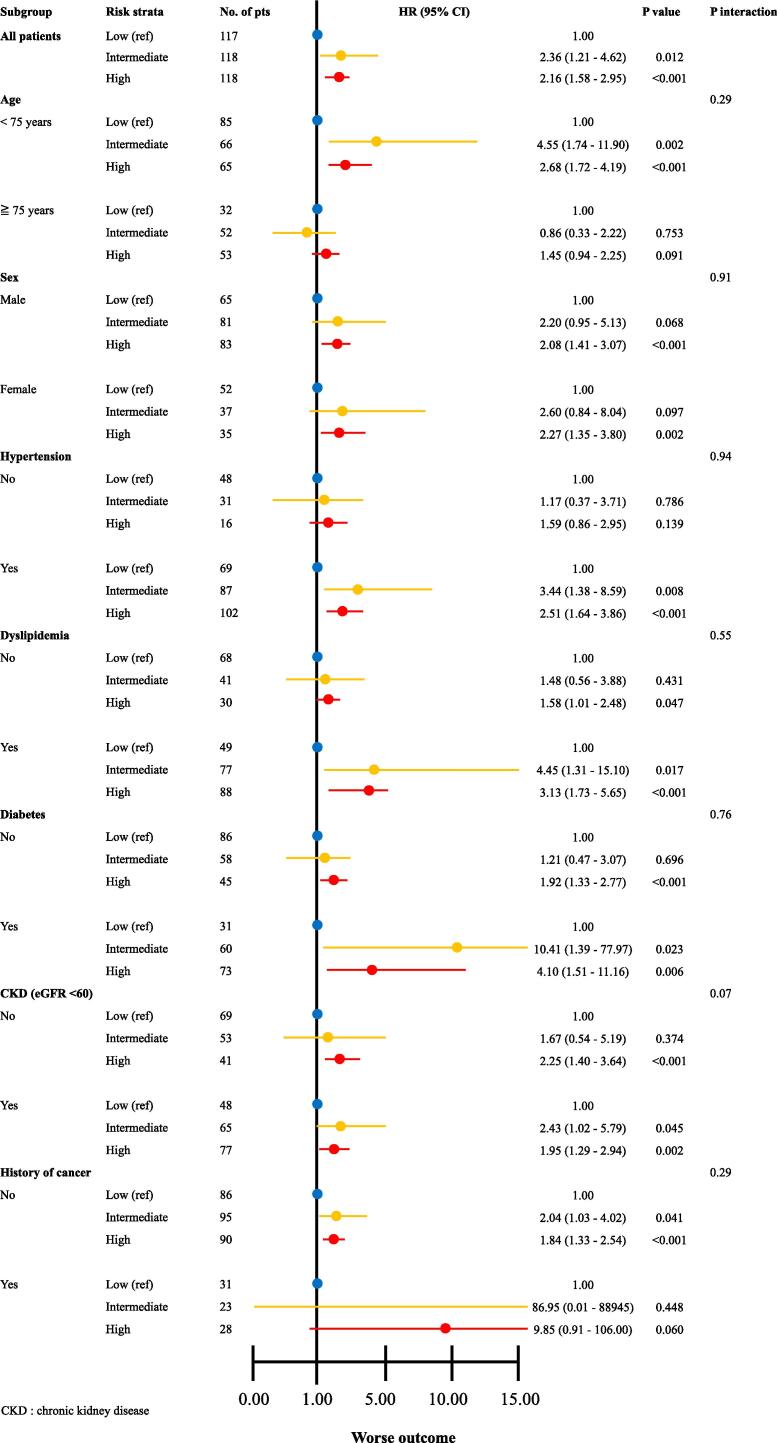
Hazard ratios for the primary endpoint between the three score strata according to clinical subgroups.

The Agatston score was also applied to the clinical subgroups identified according to patient characteristics. The Cox proportional hazards model for the primary endpoint among the three score strata confirmed the usefulness of the score determined using non-ECG-gated CT across various subgroups (Fig. 2). However, it is important to recognize the potentially significant impact of limited sample sizes within certain subgroups on the robustness of these findings. Smaller sample sizes can increase the variability of the results, making definitive conclusions difficult. Additionally, potential confounding factors that were not fully addressed in the analyses should be carefully considered when interpreting these results.

### 3.4 Long-term outcomes and factors correlated with the secondary endpoint

During the median follow-up period of 19.2 (IQR, 2.6–39.7) months, 81 patients (22.9 %) reached the secondary endpoint, which included 24 non-cardiovascular deaths, 22 cases of cancer development, and 35 cases of hospitalization for a worsening non-cardiovascular event including infection (n = 25) and bleeding complications (n = 10). The event rates were 85.9 % at 1 year, 71.8 % at 3 years, and 52.1 % at 5 years. Kaplan–Meier analysis revealed prognostic differences among patients with low, intermediate, and high Agatston scores throughout the follow-up period (Fig. 1B). In particular, a large increase in the event rate was observed between the low and intermediate score groups.

As illustrated in Fig. 1A and B, censoring was performed at the time of the primary and secondary endpoints.

## 4 Discussion

The Agatston score obtained using non-ECG-gated CT was strongly correlated with cardiovascular events. Moreover, the score severity effectively stratified risk, as demonstrated through various subgroup analyses. A remarkable association was also observed with non-cardiovascular events.

The Agatston score is a valuable prognostic indicator for cardiovascular events [17], [18], traditionally defined by ECG-gated CT and primarily used to detect coronary diseases. However, non-ECG-gated CT offers considerable versatility in various clinical scenarios, making it an excellent tool for screening [19]. Historically, investigating Agatston scores using non-ECG-gated CT has been challenging due to issues such as motion artifacts. However, advancements in deep learning analytical methods are being explored to develop more accurate indicators [20]. These advanced applications are not widely available or feasible in all areas, but our results suggest that the current approach can effectively predict long-term events without relying on these technologies.

Importantly, non-ECG-gated CT scans are easy to obtain, and the Agatston score can be calculated using commonly available applications. This accessibility enhances the potential for practical application of non-ECG-gated CT in clinical settings. Furthermore, we observed that the Agatston score determined using non-ECG-gated CT was correlated not only with cardiovascular events but also with non-cardiovascular events. These findings suggest that high scores, as determined using non-ECG-gated CT, can serve as a surrogate marker for worsening events, highlighting the potential of non-ECG-gated CT as a valuable tool for prognostic assessment in various clinical applications. Our findings indicate that the Agatston score is associated with non-cardiovascular events, including hospitalization for infection. A previous study reported an association between high Agatston scores and an increased risk of severe infectious diseases, indicating a tendency toward a higher incidence of infections [21]. Another study examined the association between Agatston scores and cancer prognosis and found a tendency toward a higher risk of cancer development [22]. In our study, the risks for both cardiovascular and non-cardiovascular events tended to increase with higher Agatston scores. In cases of intermediate to severe scores, proactive measures are necessary to prevent infections, facilitate early detection of malignancies, and manage cardiovascular events.

### 4.1 Limitations

This study had some limitations. First, its observational and retrospective nature means prevents us from establishing a cause–effect relationship between endpoints and Agatston scores. Furthermore, the follow-up periods varied among patients, and management decisions were left to the discretion of each attending physician. Second, it was conducted at a single institution and was limited to patients who underwent coronary angiography, likely introducing selection bias and other institution-specific biases. The selection criteria may have affected the cohort characteristics, potentially affecting the generalizability of our findings. Third, the Agatston score may have been affected by motion artifacts due to the use of non-ECG-gated CT scans. This is a significant concern because motion artifacts can lead to inaccuracies in the coronary artery calcium assessment, potentially affecting the reliability of the Agatston score. Another issue is that the images were acquired using a 5-mm slice thickness, so the Agatston score required correction. We should also note that non-gated CT may have missed mild coronary calcifications, further complicating the interpretation of the results, and glycemic control was managed at the discretion of the individual physicians, potentially introducing variability in the interventions and biasing the outcomes. More research is needed to explore the relationship between glycemic control and coronary CT findings, and standardized methodologies should be implemented. Finally, it will be necessary to address concerns related to motion artifacts and their effects on the accuracy of non-ECG-gated CT assessments to ensure the validity of findings in this area.

## 5 Conclusion

The Agatston score obtained from non-ECG-gated CT can predict both cardiovascular and non-cardiovascular events. This method is easy to perform and is widely used for screening various diseases, and can help identify high-risk patients early. However, motion artifacts may affect the accuracy of the Agatston score in these scans. Additionally, the retrospective nature of this study may have introduced biases. Therefore, future prospective multicenter studies are needed to validate these findings and further explore the predictive value of the Agatston score in non-ECG-gated CT for both types of events.

## Ethical statement

The study protocol was approved by the Ethics Committee of Akita University Graduate School of Medicine (approval number: 2740) and was conducted according to the tenets of the 1975 Declaration of Helsinki. The requirement for informed written consent was waived for all participants due to the retrospective nature of the study, and participants had the option to withdraw from the study via our website.

## CRediT authorship contribution statement

**Tomitaka Wakaki:** Writing – review & editing, Writing – original draft, Visualization, Validation, Software, Resources, Project administration, Methodology, Investigation, Funding acquisition, Formal analysis, Data curation, Conceptualization. **Yusuke Takagi:** Methodology. **Yuto Ono:** Data curation. **Ryosuke Kato:** Data curation. **Ken Abe:** Data curation. **Hiroyuki Watanabe:** Writing – review & editing, Supervision, Conceptualization.

## Funding

This research did not receive any specific grant from funding agencies in the public, commercial, or not-for-profit sectors.

## Declaration of competing interest

The authors declare that they have no known competing financial interests or personal relationships that could have appeared to influence the work reported in this paper.
